# Comparison of Rituximab Originator With CT-P10 Biosimilar in Patients With Primary Sjögren's Syndrome: A Retrospective Analysis in a Real-Life Setting

**DOI:** 10.3389/fmed.2020.00534

**Published:** 2020-09-08

**Authors:** Viktoriya Pavlych, Claudia Di Muzio, Alessia Alunno, Francesco Carubbi

**Affiliations:** ^1^Rheumatology Unit, Department of Biotechnological and Applied Clinical Science, School of Medicine, University of L'Aquila, L'Aquila, Italy; ^2^Rheumatology Unit, Department of Medicine, University of Perugia, Perugia, Italy; ^3^COVID-19 Medical Unit, Department of Medicine, ASL1 Avezzano-Sulmona-L'Aquila, San Salvatore Hospital, L'Aquila, Italy

**Keywords:** primary Sjögren's syndrome, rituximab, ESSDAI, ESSPRI, biosimilar

## Abstract

**Introduction:** Over the last two decades, rituximab (RTX) has been widely used, albeit off-label, in primary Sjögren's syndrome (pSS). Several studies reported that B lymphocyte depletion with RTX is effective to treat some aspects within the disease spectrum, by reducing disease activity and affecting the inflammation and lymphoid organization that occur in target tissues. Notwithstanding, randomized controlled trials failed to confirm such evidence. With the recent release of several RTX biosimilars on the market, their efficacy and safety compared to the originator must be ascertained across different indications. This study aimed at comparing efficacy and safety of RTX originator and CT-P10 RTX biosimilar in pSS patients in a real-life setting.

**Methods:** Clinical and laboratory records of pSS patients referring to a tertiary rheumatology clinic were retrospectively evaluated. Patients having received at least two courses of either RTX originator or CT-P10 with complete data at baseline and after 12, 24, 36, and 48 weeks of treatment were enrolled. Disease activity was assessed with the EULAR Sjögren's Syndrome Disease Activity Index (ESSDAI) and its clinical version without the biological domain (clinESSDAI). Patient-reported symptoms were assessed with the EULAR Sjögren's Syndrome Patient-Reported Index (ESSPRI). Adverse events (AEs) occurring during the study period were also recorded.

**Results:** Nine patients who received RTX originator and eight patients who received CT-P10 were enrolled. Baseline clinical and serological features, including ESSDAI and ESSPRI, were similar in the two treatment groups. An efficient depletion of circulating CD19^+^ B lymphocytes was achieved in both treatment arms. Both RTX originator and CT-P10 significantly reduced ESSDAI and clinESSDAI by week 24, and no difference between the groups was observed at any timepoint. Conversely, changes of ESSPRI overtime did not differ between the two treatment arms and were not statistically significant compared to corresponding baseline values. With regard to safety, at 48 weeks of follow-up, only four mild AEs (two in the RTX originator and two in the CT-P10 group) were observed.

**Conclusion:** Our study provides the first evidence that, at 48 weeks of follow-up, RTX originator and CT-P10 display similar efficacy and safety profiles in pSS.

## Introduction

Primary Sjögren's syndrome (pSS) is a systemic autoimmune disease characterized by mucosal dryness in the majority of patients. General symptoms such as fatigue, weight loss, and fever, as well as extraglandular manifestations involving musculoskeletal system, skin, peripheral and central nervous system, kidneys, and lungs, occur in at least one-third of patients, increasing health care costs and affecting the quality of life (1–3). The evolution into B-cell lymphoma represents one of the main causes of decreased survival in pSS and occurs in about 5% of patients (4).

B cells play a major role in the pathogenesis of pSS via antibody-dependent and -independent mechanisms, and their hyperactivity, along with salivary gland infiltration and development of B-cell follicles containing germinal center–like structures, represents the hallmarks of the disease (4–7).

Therapeutic management of pSS is based on symptomatic treatment of sicca symptoms and broad-spectrum immunosuppression for systemic disease, but data concerning efficacy and safety of the therapeutic options available are often insufficient (8). Although the emergence of biological therapies has increased the therapeutic armamentarium available to treat pSS, their use in clinical practice is limited by the lack of licensing (9). Given the central role of B cells in pSS pathogenesis, a B-cell targeting therapy represents an unarguable and intriguing therapeutic approach in this disease. Rituximab (RTX) is a chimeric murine/human IgG1 monoclonal antibody (with human kappa and IgG1 constant regions and murine light- and heavy-chain variable regions) targeting the CD20 molecule (human B lymphocyte–restricted differentiation antigen, Bp35) found on the surface of most B cells, including pre-B, mature B lymphocytes, and malignant B cells, but not on stem cells, pro–B cells, normal plasma cells, or other normal tissues. There are at least four postulated mechanisms of action for RTX: complement-mediated cytotoxicity, antibody-dependent cell-mediated cytotoxicity, induction of apoptosis, and saturation of the Fc receptors of effector cells, and all of them may contribute to the therapeutic effect in pSS (10, 11).

In pSS, RTX has been tested in four randomized controlled trials (RCTs) (12–15), three prospective cohort studies (16–18), and one case–control study (19). It is evident from most studies that RTX has a positive impact on B-cell numbers and activity, both in the peripheral blood and in salivary glands, but the clinical efficacy of B-cell depletion therapy with RTX in pSS remains controversial (20). In particular, although the majority of studies showed efficacy in at least one of the systemic outcomes analyzed, such as global response, organ-specific response, EULAR Sjögren's Syndrome Disease Activity Index (ESSDAI) reduction, and prednisone reduction, the evidence reported by RCTs is weak. Moreover, an analysis of data from The Trial of Anti–B cell Therapy in Patients With Primary Sjögren's Syndrome shows that RTX is not cost-effective (15). Therefore, the recent EULAR recommendations for the management of Sjögren's syndrome pointed out that the use of RTX should be reserved to selected patients with severe, refractory systemic disease (8).

RTX was the first monoclonal antibody to be approved for the treatment of some type of blood cancer and inflammatory conditions as rheumatoid arthritis, granulomatosis with polyangiitis, microscopic polyangiitis, and pemphigus vulgaris [MabThera® [Roche, Welwyn Garden City, United Kingdom] in Europe and Rituxan® [Genentech, San Francisco, CA] in the USA] (21, 22). As this anti-CD20 monoclonal antibody has now reached patent expiry date, biosimilar versions are in development. In particular, CT-P10 [Truxima® (Celltrion Incheon, Republic of Korea)] has recently been approved in Europe for all indications held by MabThera® (23).

Biosimilars have the potential to broaden patient access to biologics and reduce the economic burden of health care systems. During the development of a biosimilar, data that directly compare the proposed biosimilar with the reference product are required. These comparative data are generated in a stepwise hierarchical process from extensive laboratory-based structural analyses and functional assays, to clinical safety and efficacy (24). Demonstration of similarity in these trials, along with extensive evidence of similarity from other tests, allows the extrapolation of data with the reference compound to the biosimilar in other non-tested indications (25).

Several clinical trials assessing CT-P10 in follicular lymphoma and rheumatoid arthritis (26–33) and a few data concerning the real-world experience (34, 35) have been published so far. However, data concerning this compound in pSS are not available.

To our knowledge, no study has compared efficacy and safety of any approved RTX biosimilar to RTX originator in pSS patients. Therefore, the aim of this retrospective, observational, single-center study was to compare the efficacy and safety of CT-P10 (Truxima®) with RTX originator (MabThera®) in a real-life cohort of patients with pSS.

## Materials and Methods

### Study Population

This was a retrospective, observational, single-center study of pSS patients receiving RTX off-label in a tertiary Rheumatology Unit (ASL1 Avezzano-Sulmona-L'Aquila and University of L'Aquila, Italy). We included 17 consecutive patients with pSS diagnosis, with a disease duration of < 5 years and a systemic moderate–high activity (ESSDAI ≥5) (36) who received the first RTX infusion between December 2013 and January 2019. Patients receiving the first infusion between December 2013 and November 2017 received RTX originator (MabThera®) (nine patients), and those initiating RTX after November 2017 received CT-P10 (Truxima®) (eight patients), because of local health policy regulations. Patients with secondary Sjögren's syndrome; severe cardiac, pulmonary, renal or hematologic failure; a history of cancer in the last 5 years; hepatitis B or hepatitis C infection, human immunodeficiency virus infection, tuberculosis, severe diabetes, and any other chronic disease; or evidence of infection; and if they were unable to understand and to adhere to the treatment were not eligible to start RTX and therefore excluded from this study. Treatment with conventional synthetic disease-modifying antirheumatic drugs (csDMARDs) and corticosteroids was allowed during the study period because of the moderate–high disease activity. However, any change in the dose or schedule was noted.

### RTX Administration

Patients received infusion of 1,000 mg of either RTX originator or CT-P10 at day 1 and at day 15 to complete a course of therapy. This course was repeated after 24 weeks. During the study period, all patients received two courses of therapy with RTX (baseline and week 24). To minimize adverse effects, all patients were pretreated with methylprednisolone (40 mg intravenously), paracetamol (1,000 mg orally), and chlorpheniramine (10 mg intravenously).

### Clinical and Laboratory Evaluation

Clinical and laboratory evaluations were performed at baseline (W0), week 12 (W12), week 24 (W24), week 36 (W36), and week 48 (W48) of treatment. The disease activity was assessed using the ESSDAI and clinical ESSDAI (clinESSDAI), whereas patient-reported outcomes (PROs) included the EULAR Sjögren's Syndrome Patient-Reported Index (ESSPRI) and the patient's assessment of general health (GH) score [on a 0–100-mm visual analog scale (VAS) with “very poor” and “very well” as anchors]. We also considered the reduction in the daily dose of prednisone. Total lymphocyte count, CD19^+^ B lymphocytes, serum γ-globulins, immunoglobulin classes (IgG, IgA, and IgM) concentration, and serum rheumatoid factor (RF) were regularly measured. Patients were asked to report the occurrence of systemic or local adverse events (AEs) related to treatment at each visit. AEs were judged as serious if they resulted in death, were life-threatening according to the investigator's own judgment, caused hospital admission, resulted in birth defect (from unplanned pregnancies) or disability, or were important medical events that could have jeopardized the patient or needed intervention to prevent another serious AE, or both.

### Study Endpoints

The primary efficacy endpoint of this study was the delta (Δ) ESSDAI, clinESSDAI, and ESSPRI achieved by CT-P10 compared with RTX originator. The primary safety outcome was the number of AEs during the study period. Secondary exploratory endpoints included the percentage of patients achieving a minimal clinically important improvement (MCII) with ESSDAI (drop of at least three points), MCII with ESSPRI (drop of at least one point or 15% of baseline value), and patient acceptable symptom state (PASS) (ESSPRI <5) at W48, laboratory measures, and GH.

### Statistical Analysis

IBM SPSS Statistics 23.0 software was used for statistical analysis. One-way analysis of variance and multiple-comparisons *post hoc* tests were employed to calculate differences between baseline and following timepoints. Differences between the demographic and clinical characteristics of the two treatment groups at baseline and between the two treatment arms at each timepoint were tested with non-parametric Mann–Whitney *U*-test. When required, χ^2^ test was also employed. *P* < 0.05 were considered significant.

## Results

### Baseline Demographic and Clinical Characteristics

Demographic and clinical characteristics of the two treatment groups at baseline are shown in Table 1. We included 17 patients: nine in the RTX originator group and eight in the CT-P10 group. We did not observe any significant differences in age, gender, and disease duration, or in clinical and laboratory parameters. None of the included patients displayed B-cell lymhoproliferative disease. The proportion in the use of the different csDMARDs and corticosteroids did not differ in both groups. In detail, three patients were in treatment with methotrexate, one with leflunomide, and six with hydroxychloroquine. Eight patients in the RTX originator group and five patients in the CT-P10 group were also in treatment with prednisone (mean dosage at baseline 6.3 and 4.2 mg, respectively). All 17 patients completed the 12 months' follow-up and are still in treatment.

**Table 1:** Demographic and clinical characteristics of patients at baseline.

	**CT-P10**	**RTX originator**	***p*-value**
	**n (%)**	**n (%)**	
**Number**	8	9	—
**Age, mean (SD), years**	60.1 (9.1)	51.8 (12.8)	ns
**Female gender, n (%)**	7 (87)	8 (89)	ns
**Disease duration, mean (SD), years**	1.29 (1.5)	1.75 (1.6)	ns
**Focus score ≥ 1, n (%)**	8 (100)	9 (100)	ns
**Xerostomia, n (%)**	8 (100)	8 (89)	ns
**Xerophthalmia, n (%)**	8 (100)	9 (100)	ns
**Salivary gland enlargement, n (%)**	1 (12)	3 (33)	ns
**ESSDAI, mean (SD)**	12.0 (7.3)	12.6 (6.6)	ns
**ClinESSDAI, mean (SD)**	11.4 (7.1)	11.4 (6.4)	ns
**ESSDAI domains, n (%)**			
Constitutional	1 (12)	6 (67)	ns
Lymphoadenopathy	3 (37)	3 (33)	ns
Glandular	1 (12)	3 (33)	ns
Articular	8 (100)	8 (89)	ns
Cutaneous	1 (12)	1 (11)	ns
Pulmonary	1 (12)	1 (11)	ns
Renal	0 (0)	0 (0)	ns
Muscular	0 (0)	0 (0)	ns
PNS	3 (37)	3 (33)	ns
CNS	1 (12)	0 (0)	ns
Hematological	0 (0)	2 (22)	ns
Biological	4 (50)	7 (78)	ns
**Hypergammaglobulinemia, n (%)**	3 (37)	5 (55)	ns
**Reduced complement fractions, n (%)**	2 (25)	3 (33)	ns
**Autoantibodies, n (%)**			
Neither anti-Ro nor anti-La	3 (37)	5 (55)	ns
Anti-Ro only	4 (50)	2 (22)	ns
Anti-Ro and anti-La	1 (12)	2 (22)	ns
RF	5 (62)	4 (44)	ns
**ESSPRI, mean (SD)**	6.8 (2.1)	7.8 (2.0)	ns
**GH, mean (SD)**	30 (16.0)	33.3 (22.9)	ns
**csDMARDs, n (%)**	4 (50)	4 (44)	ns
**Prednisone, n (%)**	5 (62)	8 (89)	ns

*RTX, rituximab; SD, standard deviation; ns, not statistically significant; ESSDAI, EULAR Sjögren's syndrome disease activity index; PNS, peripheral nervous system; CNS, central nervous system; RF, rheumatoid factor; ESSPRI, EULAR Sjögren's Syndrome Patient-Reported Index; GH, global health; csDMARDs, conventional synthetic disease-modifying antirheumatic drugs*.

### Primary Endpoints

**Figure 1:**
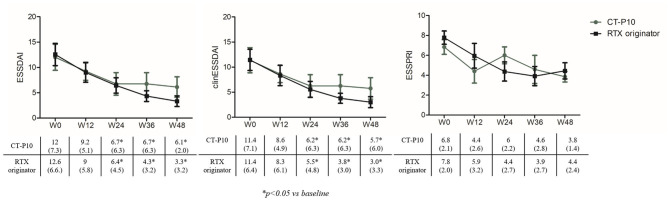
EULAR Sjögren's Syndrome Disease Activity Index (ESSDAI), clinical (clin) ESSDAI, and EULAR Sjögren's Syndrome Patient-Reported Index (ESSPRI) during the study period in the two treatment arms. RTX, rituximab; W, week.

Figure 1 shows ESSDAI, clinESSDAI, and ESSPRI across the study period. In both treatment groups, ESSDAI and clinESSDAI started to decrease by W12, albeit significantly only from W24. The significant difference with baseline was consistent up to W48; at none of the timepoints the ΔESSDAI and ΔclinESSDAI were significantly different between the two treatment arms (Table 2). At W48, MCII with ESSDAI were achieved by all patients regardless of the treatment arm. Table 3 shows the improvement within each ESSDAI domain in the two treatment groups at W48. Improvement was predominantly seen in constitutional, lymphoadenopathy, glandular, articular, hematological, and biological domains, and still the two cohorts did not display any difference in this regard. In addition, all the five patients (three in the originator group and two in the CT-P10 group) with reduced complement fractions at baseline still displayed it at W48. With regard to ESSPRI, neither CT-P10 nor RTX originator was able to significantly reduce it in line with previous studies (Figure 1), and no difference could be observed with regard to the ΔESSPRI between the two treatment groups at any timepoint (Table 2). However, MCII with ESSPRI (reduction of at least one point or reduction of 15% of baseline value) was achieved at W48 by six patients (75%) in the CT-P10 group and all nine patients (100%) in the RTX originator group (not statistically significant). PASS was achieved by a similar proportion of patients in the two treatment groups (CT-P10 group: *n* = 4; 50%; RTX originator: *n* = 4; 44%; not statistically significant) at W48.

**Table 2:** Change of disease activity and patient-reported symptoms at the different timepoints compared to baseline.

	**W12**		**W24**		**W36**		**W48**	
	**CT-P10**	**RTX originator**	**CT-P10**	**RTX originator**	**CT-P10**	**RTX originator**	**CT-P10**	**RTX originator**
ΔESSDAI, mean (SD)	−2.75 (2.37)	−3.00 (2.18)	−5.12 (2.10)	−5.89 (4.99)	−5.12 (2.10)	−7.67 (5.59)	−5.62 (2.26)	−8.44 (5.03)
ΔclinESSDAI mean (SD)	−2.75 (2.37)	−3.11 (2.26)	−5.12 (2.10)	−5.89 (4.99)	−5.12 (2.10)	−7.67 (5.59)	−5.62 (2.26)	−8.44 (5.03)
ΔESSPRI mean (SD)	−4.1 (4.4)	−3.15 (3.1)	−1.6 (2.3)	−3.3 (2.9)	−4.5 (2.9)	−4.3 (2.6)	−3.46 (2.9)	−3.33 (0.91)

*RTX, rituximab; ESSDAI, EULAR Sjögren's Syndrome Disease Activity Index; clin, clinical; ESSPRI, EULAR Sjögren's Syndrome Patient-Reported Index*.

**Table 3:** ESSDAI domains in the study cohort at W48.

	**CT-P10 n (%)**	**RTX originator n (%)**	***p*-value**
**Number**	8	9	—
**ESSDAI domains**			
Constitutional	0 (0)	0 (0)	ns
Lymphoadenopathy	1 (12)	0 (0)	ns
Glandular	0 (0)	1 (11)	ns
Articular	6 (75)	5 (55)	ns
Cutaneous	0 (0)	0 (0)	ns
Pulmonary	1 (12)	1 (11)	ns
Renal	0 (0)	0 (0)	ns
Muscular	0 (0)	0 (0)	ns
PNS	3 (37)	2 (22)	ns
CNS	1 (12)	0 (0)	ns
Hematological	0 (0)	0 (0)	ns
Biological	2 (25)	2 (22)	ns

*RTX, rituximab; ESSDAI, EULAR Sjögren's Syndrome Disease Activity Index; ns, not statistically significant; PNS, peripheral nervous system; CNS, central nervous system*.

As far as the safety is concerned, a total of four AEs were recorded in four patients across the 12 months' follow-up: one mild cutaneous reaction (RTX originator group, T6) and three upper airways infections (CT-P10 group, one at T3 and one at T9; RTX originator group, T6), which required antibiotic therapy.

### Secondary Clinical and Laboratory Outcomes

**Figure 2:**
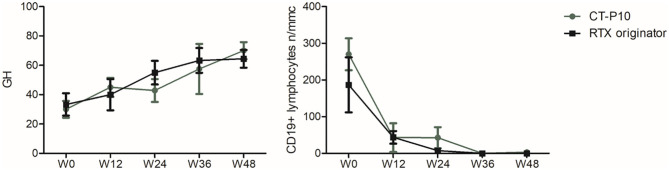
Global health (GH) and CD19^+^ lymphocyte count during the study period in the two treatment arms. W, week.

GH showed a trend toward improvement starting from W12 in both groups and becoming significant at W48 compared to baseline, with no significant differences in the two groups (RTX originator group W0: 33.3 ± 22.9, W48: 70 ± 17.89; *p* = 0.01; CT-P10 group W0: 30 ± 16, W48: 76.7 ± 11.5; *p* = 0.02) (Figure 2). Both RTX originator and CT-P10 effectively depleted circulating B lymphocytes as demonstrated by the serial measurement of CD19^+^ cells (Figure 2). The total lymphocyte count, however, remained unaffected (data not shown). Serum γ-globulins, immunoglobulin classes (IgG, IgA, and IgM) and RF were regularly monitored and did not show significant changes overtime (data not shown).

The majority of patients taking corticosteroids at baseline tapered the dose until withdrawal with only two out of five patients in the CT-P10 group and five out of eight patients in the RTX originator group still on treatment (mean dose 3 mg in both groups).

## Discussion

This is the first study demonstrating that CT-P10 and RTX originator show similar efficacy and safety profiles in pSS patients with moderate–high disease activity and disease duration of < 5 years at 48 weeks of follow-up. Concerning the clinical efficacy, in both treatment groups, ESSDAI and clinESSDAI started to slowly decrease by W12, with a significant difference with baseline starting from W24 and remaining sustained up to W48. Furthermore, we did not observe any significant differences in the ΔESSDAI, ΔclinESSDAI, and MCII with ESSDAI between the two treatment arms. Interestingly, this improvement mirrored the dose reduction of prednisone, with its withdrawal in three patients in both groups at W48. In addition, in our cohort, the ESSDAI domains were mainly represented by articular, lymphoadenopathy, and peripheral nervous system involvement in both groups and constitutional in originator. Therefore, our study confirms existing evidence that RTX may be considered a valuable therapeutic option in patients with this specific phenotype. However, despite the generally acknowledged beneficial effects of RTX treatment on biological parameters, clinical outcomes vary between studies (20). Available data on the systemic efficacy of RTX in pSS come from large studies that included more than 400 patients, and the predominant regimen of administration was two doses of 1,000 mg each administered 15 days apart (8, 9). The great majority of these studies showed efficacy in at least one of the systemic outcomes analyzed, considering global response, organ-specific response, ESSDAI reduction, or prednisone reduction, thereby providing a reasonable rationale for the use of this therapy in specific clinical settings. These concepts have been incorporated in the recent EULAR recommendations for the management of Sjögren's syndrome with topical and systemic therapies that reserve the use of RTX to patients with severe refractory disease (8, 9). PROs with VAS have been used in most studies, including two recent large RCTs (14, 15) to assess subjective symptoms. Devauchelle-Pensec et al. (14) found no significant results in the primary outcome (≥30-mm improvement at week 24 on at least two out of four VAS scores—dryness, fatigue, pain, global, 23 vs. 22%; *p* = 0.91), whereas Bowman et al. (15) found no significant results in the primary outcome (reduction ≥ 30% at week 48 in either fatigue or oral dryness VAS, RTX 39.3 vs. placebo 36.8%; *p* = 0.76). This is in line with our findings revealing a failure in achieving a significant reduction of ESSPRI up to W48. Of interest, despite a considerable proportion of patients reached MCII with ESSPRI at W48, namely, a reduction of at least one point or 15% reduction of baseline value, approximately only half of them in each treatment group achieved a PASS, namely, an ESSPRI of < 5. In our study, both treatments also induced a similar improvement of GH, which based on the ESSDAI and ESSPRI values overtime seemed to be more related to systemic disease activity rather than sicca symptoms, fatigue, or pain.

The number of patients underreporting or overreporting their symptoms may influence the results of studies and underscores the uniqueness of individual perception of pSS-related symptoms regardless of disease activity and therefore showing different response patterns to RTX treatment. In this regard, the discrepancy between PROs, objective measurement of glandular function, and systemic disease activity indexes in pSS underpins that they represent complementary perspectives to obtain a holistic view of the individual, and therefore, all of them should be explored and implemented in clinical practice (37). With regard to the effect on B cells, we confirmed that treatment with RTX leads to a nearly complete depletion in the peripheral blood, without any significant differences between the two treatment groups (12, 16). Although we did not observe any change in serum RF, γ-globulins, and immunoglobulin classes, it has been postulated that by interfering with B-cell activation likely one aspect contributing to the amelioration of systemic disease activity in pSS patients may be the lower levels of autoantibodies and proinflammatory cytokines (38). CT-P10 was the first RTX biosimilar (39) and could represent a cost-effective and safe therapeutic alternative to RTX originator, possibly facilitating access to therapy for pSS patients with severe, refractory systemic disease.

Budget impact analysis models estimated the expected changes in expenditure that would occur as a result of the adoption of a new therapeutic intervention. On this basis, it was demonstrated that introduction of CT-P10 could be associated with significant budget savings in European Union countries, for both in-label and off-label indications (40). With the expiry date of patent protection approaching for several originator biologic DMARDs, we have witnessed the development of less expensive competitor products of sufficient similarity, called biosimilars. Regulatory approval is based on the totality of evidence for biosimilarity derived from a comprehensive comparability exercise with the reference medicine (41). This comparability exercise includes extensive physicochemical and structural evaluations, as well as data from preclinical and clinical pharmacokinetic, pharmacodynamic, and immunogenicity assessments. The final step in the development process is confirmatory phase III clinical trial in patients with the specific disease. However, in line with regulatory requirements, approval of CT-P10 in some indications of RTX was based in part on the extrapolation of clinical data collected in other indications, plus a scientific justification based on the consistency of RTX mechanisms of action across indications (42–44). Although there are several discrepancies in assessing and reporting immunogenicity data of biosimilars for CD20 inhibitors, data collected in trials confirmed that immunogenicity parameters of CT-P10 were similar to those of its reference product (26, 27, 30). Furthermore, it was reassuring to observe that the safety profile of CT-P10 was similar to RTX originator also in pSS, with upper airway infections being the most frequent AEs in both groups.

We acknowledge that this study displays some limitations including the retrospective nature, the heterogeneity of clinical spectrum and disease duration, and the small number of patients. In addition, treatment choice between RTX originator and CT-P10 depended on the time of the first infusion and not the physician's decision, because of local health policy issues. Notwithstanding, we believe that given the lack of studies on CT-P10 in pSS, it provides important insight to clinicians who will be required to use this compound in pSS patients.

## Conclusion

RTX originator is a cornerstone in therapeutic strategies for rheumatic diseases, including pSS; however, economic issues may be a major barrier to access the best available care, particularly in some countries. Therefore, the use of biosimilars can only be expected to increase. Whether the use of biosimilars will be of greater benefit from a societal perspective will depend on their cost-effectiveness and safety. In this perspective, the collection of efficacy and safety data for all in-label and off-label indications is of paramount importance to identify and tackle potential differences among compounds and ultimately does not affect the quality of care for patients with pSS.

## Data Availability Statement

All datasets generated for this study are included in the article/supplementary material.

## Ethics Statement

Ethical review and approval was obtained in accordance with local legislation and institutional requirements. The patients/participants provided their written informed consent to participate in this study.

## Author Contributions

All authors drafted and approved the final version of the manuscript.

## Conflict of Interest

The authors declare that the research was conducted in the absence of any commercial or financial relationships that could be construed as a potential conflict of interest. The reviewer EB declared a shared affiliation, though no other collaboration, with one of the authors AA to the handling editor.
